# Cumulative and Legacy Effects of Irrigation on Growth, Physiology, and Gene Expression in *Veronica longifolia* Under Field Conditions

**DOI:** 10.1002/pei3.70202

**Published:** 2026-09-03

**Authors:** Jin‐Woo Kim, Ji Hun Yi, Sang Heon Kim, Ji Young Jung, Ki Yeol Jang, Young Ji Kim, Oh Yoon Kwon, Wonwoo Cho, Wan Soon Kim

**Affiliations:** ^1^ Forest Biological Resources Utilization Center Korea National Arboretum Yangpyeong South Korea; ^2^ Department of Environmental Horticulture University of Seoul Seoul South Korea

**Keywords:** carryover response, climate change, cumulative effects, irrigation history, physiological regulation, seasonal response, transcriptional response

## Abstract

Climate change is expected to increase the frequency of fluctuating moisture regimes on the Korean Peninsula, yet plant responses to moderate field moisture variation remain poorly understood. We investigated the effects of irrigation on growth, physiology, and gene expression of the native perennial plant *Veronica longifolia* under field conditions. Plants were exposed to irrigated and nonirrigated treatments for 20 weeks in 2024, followed by a subsequent growing season in 2025 without irrigation to assess cumulative and carryover effects. During the 2024 treatment period, seasonal mean soil water content ranged from 14.4% to 27.6% in the irrigated plot and from 10.1% to 24.8% in the nonirrigated plot, remaining within or above a generally favorable range for growth and suggesting that severe drought stress was unlikely. Nevertheless, irrigation increased plant height and leaf area, with leaf area 31.1% and 28.7% greater in the irrigated plot during the rainy and postrainy periods, respectively. Irrigation also enhanced reproductive performance, increasing seed mass, seed length, and seed width by 16.7%, 14.4%, and 14.2%, respectively. Seasonal physiological responses showed minimal differences in photosynthetic parameters, except for transient reversals during the rainy season, likely associated with excessive soil moisture. In 2025, biomass differences between treatments disappeared under uniform nonirrigated conditions; however, plants previously subjected to irrigation maintained higher photosynthetic activity and stomatal conductance. Gene expression analyses showed treatment‐history‐associated differences in transcripts commonly linked to abiotic stress regulation (*VlADH, VlDREB1*, and *VlERF*), antioxidant defense (*VlPOD*), carbon assimilation (*VlrbcS*), and photosystem II (PSII) repair (*VlpsbA*). Because gene expression was assessed at a single sampling time, these transcriptional patterns should be interpreted as possible carryover responses associated with prior irrigation history rather than direct evidence of physiological memory mechanisms. These findings suggest that moderate differences in field moisture availability can be associated with cumulative growth responses within the treatment year and with physiological and transcriptional differences in the following year.

## 1 Introduction

Climate change represents one of the greatest challenges facing humanity. Global mean surface temperature has increased by approximately 1°C relative to 1850–1900 (Lindsey and Dahlman 2024), with much of the warming occurring in recent decades. Climate change now regularly manifests worldwide as extreme events, with severe consequences for ecosystems, human societies, and cultural practices (McKenzie et al. 2023). However, evidence of climate‐change impacts is strongest and most comprehensive for natural systems (IPCC 2014).

Under the RCP2.6, RCP8.5, SSP1‐2.6, and SSP5‐8.5 scenarios, mean temperature on the Korean Peninsula is projected to increase by 1.8°C, 4.7°C, 2.6°C, and 7.0°C, respectively, and precipitation by 5.5%, 13.1%, 3.0%, and 14.0%, respectively, during the late 21st century (2071–2100) relative to 1981–2010 (KMA 2018; NIMS 2020). Extreme high temperatures and heavy rainfall events are expected to become more frequent (Sung et al. 2023). As a result, precipitation will likely be more concentrated in the summer rainy season, increasing the likelihood of drought during other periods (Park et al. 2013; Sohn et al. 2014; Gwak et al. 2018; NIMS 2018). Consequently, plants on the Korean Peninsula will increasingly experience combined heat and water‐related stresses, underscoring the need for species‐specific, field‐based assessments of climate responses. Numerous studies have examined plant responses to climate change in the fields of ecology and agriculture on the Korean Peninsula (Kim et al. 2010; Kang 2013; Nam et al. 2017; Lee et al. 2020; Choi et al. 2021).

With global greenhouse gas emissions projected to rise for the foreseeable future, elevated atmospheric CO_2_ and associated shifts in temperature and precipitation are expected to alter plant ecophysiology, distributions, and interactions with other organisms (IPCC 2014). Consequently, anthropogenic climate change has generated thousands of studies on its effects on plant growth, reproduction, phenology, and distribution (Parmesan and Hanley 2015), yet relatively few examine counterintuitive or unexpected responses. In many well‐studied communities, a minority of species show no response or respond oppositely to local climate trends (Parmesan and Yohe 2003; Root et al. 2003, 2005; Rosenzweig et al. 2008; Poloczanska et al. 2013). Meta‐analyses indicate that phenological responses to experimental warming often do not match long‐term observational trends for the same species in the same regions (Wolkovich et al. 2012). Impacts occurring in a given period can also have lasting consequences, such as sustained reductions in woody plant stress tolerance (Fickle et al. 2025). Beyond immediate physiological responses, repeated differences in water availability may generate persistent effects on plant functioning, potentially influencing both metabolic and transcriptional regulation. Evidence from grapevine has shown that water‐management strategies can alter drought‐related physiological traits and stress‐responsive gene expression under open‐field conditions, suggesting that past environmental conditions may contribute to shaping subsequent plant responses (Cataldo et al. 2025). Together, these cumulative and sometimes counterintuitive effects complicate predictions of plant responses and the resilience of plant communities (Bhadra et al. 2022). Moreover, extrapolating results from controlled experiments to real‐world communities remains challenging, contributing to unresolved and sometimes contradictory findings (Parmesan and Hanley 2015). Therefore, studying seasonal responses of the same species under field and current climatic conditions is essential to improve understanding and prediction of plant responses to climate change.

The perennial herb *Veronica longifolia* is widely distributed from Europe to East Asia (Trávníček 1998). *V. longifolia* has naturally occurred in forest at elevations above 1000 m on the Korean Peninsula (An et al. 2017). However, due to its medicinal and ornamental value, it has increasingly attracted scientific attention (Oh et al. 2006; Kwon et al. 2023; Kim et al. 2024), and its utilization has gradually expanded beyond rural areas to urban gardens. Recently, interest in native plant species has intensified as they are recognized as important genetic resources for developing new horticultural cultivars capable of coping with climate change, highlighting the growing need for breeding programs aimed at climate change adaptation (Kim and Cho 2024). Studies on other native Korean plant species have investigated tolerance to various abiotic stresses, such as salinity, emphasizing the importance of understanding genetic, proteomic, and transcriptional responses underlying climate adaptation (Kim, Yi, Jeong, et al. 2025). Genetic responses of *V. longifolia* to abiotic stresses have been explored to some extent (Kim, Yi, Kim, et al. 2025). However, how *V. longifolia* responds to current climatic variability under natural field conditions, particularly across seasons, remains poorly understood. Therefore, in this study, we selected *V. longifolia* , a species with a broad global distribution, to investigate plant responses to contemporary climate conditions.

In this study, we investigated the seasonal and cumulative effects of current climatic conditions on *V. longifolia* under field conditions, comparing irrigated and nonirrigated treatments. Specifically, we aimed to (i) assess seasonal, cumulative, and including potential legacy, physiological and molecular responses to natural climatic variability, and (ii) evaluate whether supplemental irrigation alters plant responses under non‐drought conditions. “Legacy” effects were operationally defined as differences observed in 2025 under uniform nonirrigated conditions that were associated with the irrigation history imposed in 2024. This definition does not assume a specific physiological memory mechanism, but refers to measurable carryover differences in growth, physiology, or gene expression after the treatment had ceased. By doing so, this study provides insight into how native perennial plants respond to contemporary climate variability, with implications for ecological management, horticultural breeding, and climate‐resilient garden cultivation.

## 2 Materials and Methods

### 2.1 Plant Materials

*V. longifolia* used in this study was propagated from cuttings in September 2023 and maintained in the greenhouse of the Useful Plant Propagation Center, Korea National Arboretum. On April 4, 2024, individuals with 4–8 fully expanded leaves were selected and transplanted into two experimental plots (110 × 360 cm; 3.96 m^2^) in the field (37°28′45.4″ N, 127°35′57.1″ E), with 100 plants per plot. The soil consisted of naturally weathered sandy loam mixed with commercial horticultural substrate to a depth of 10 cm. Osmocote (ICL, Israel) was incorporated into the soil at 30 g·m^−1^. Transplanted plants were acclimated for two weeks, during which they were irrigated every three days. During this acclimation period, an insecticide, Straight water‐dispersible granule (sulfoxaflor; Dongbang Agro, Seoul, South Korea), was applied to control potential sucking insect pests, following the manufacturer's recommended concentration. After the 20‐week treatment period in 2024, plants were maintained in the field until aboveground growth naturally ceased. The aboveground parts were then removed, while the belowground portions were left intact in the plots. Plants were overwintered in situ under ambient field conditions without additional protection and began producing new shoots in March 2025.

### 2.2 Irrigation Treatment and Environmental Data Acquisition

Beginning on April 18, 2024, the two experimental plots were subjected to contrasting irrigation treatments. The irrigation treatment was applied at the plot level, with one irrigated plot and one nonirrigated plot. Therefore, individual plants within each plot were considered plant‐level subsamples rather than true independent experimental replicates of the irrigation treatment. The irrigated plot was irrigated daily, while the nonirrigated plot received no irrigation; on rainy days, neither plot was irrigated. The irrigation treatment lasted for 20 weeks, which were categorized into prerainy (up to week 9), rainy (weeks 10–14), and postrainy (weeks 15–20) seasons based on Korea Meteorological Administration data (KMA 2024).

During the treatment period, environmental variables—light intensity, temperature, relative humidity, and soil moisture—were continuously monitored. Soil moisture was measured using a soil moisture sensor (WT1000B, RF Sensor, Seoul, South Korea) connected to a data logger (WP700, RF Sensor, Seoul, South Korea). Temperature and relative humidity were recorded with the data logger's built‐in sensors, and light intensity was measured with external sensors (LightCount Quantum Light Sensor, Spectrum Technologies Inc., Illinois, USA) connected to a WatchDog 1000 Series Micro Station (Spectrum Technologies Inc., Illinois, USA).

Soil texture was analyzed to characterize the physical properties of the experimental soil influencing soil moisture dynamics. Soil samples were collected from the experimental plots at a depth of 0–10 cm prior to the initiation of the irrigation treatments. The samples were air‐dried, sieved through a 2‐mm mesh, and subjected to particle size analysis using standard methods (Gee and Or 2002). The soil consisted of 84.13% sand, 12.63% silt, and 3.23% clay, and was classified as sandy soil according to the USDA soil texture classification system (Soil Survey Staff 2014).

### 2.3 Measuring Plant Responses and Data Acquisition

Seasonal plant responses under the irrigation treatments were assessed at 2‐week intervals. At the end of the treatment period, plants were immediately harvested for comprehensive growth analyses. To evaluate cumulative effects of the treatments, early‐stage growth responses were measured in newly emerging shoots in April 2025, and final growth responses were assessed in August 2025. The plant growth response parameters and corresponding methodologies are summarized in Table S1.

The evaluated seasonal growth parameters included plant height, leaf area, leaf fresh and dry weights, specific leaf area (SLA), leaf water content, photosynthetic rate, stomatal conductance, maximum quantum efficiency of photosystem II (Fv/Fm), and nonphotochemical quenching (NPQ). Plant height was defined as the distance from the lowest node above the ground surface to the most distal node. Because *V. longifolia* ceases stem elongation after flowering and extends only the inflorescence, plant height after flowering was not measured. Leaf area and leaf fresh and dry weights were measured from one leaf selected from the second to fourth fully expanded leaves counted from the top. SLA was calculated as leaf area divided by leaf dry weight. Leaf water content was calculated as (fresh weight − dry weight)/fresh weight. Photosynthetic rate, stomatal conductance, Fv/Fm, and NPQ were also measured on a leaf selected from the second to fourth leaves. Plant height, photosynthetic rate, stomatal conductance, Fv/Fm, and NPQ were measured on 20 randomly selected individuals per plot. Leaf area, leaf fresh and dry weights, SLA, and leaf water content were measured on 10 randomly selected individuals per plot.

To assess aboveground and belowground biomass responses, 10 individuals randomly selected from each plot were harvested immediately after the irrigation treatment ended. The aboveground and belowground portions were separated at the lowest node of the stem. Aboveground and belowground fresh and dry weights, plant water content, mean inflorescence length, inflorescence number, and seed length, width, and 100‐seed weight were recorded. Mean inflorescence length per individual was calculated by dividing the total length of all inflorescences by the number of inflorescences. The 100‐seed weight was determined from five independent samples, each consisting of 100 seeds (total of 500 seeds). Seed length and width were measured from 10 randomly selected seeds per plot.

To evaluate the cumulative, including potential legacy effects of irrigation under the current climatic conditions, experimental plots were maintained after the irrigation treatment, and plant growth responses were assessed the following year. In April 2025, early growth responses were measured by recording the date of shoot emergence, photosynthetic rate, and stomatal conductance; shoot emergence was defined as the stage at which at least two true leaves were fully expanded. In August 2025, 10 individuals were randomly selected to evaluate aboveground and belowground fresh and dry weights, plant water content, biomass allocation ratios, photosynthetic rate, stomatal conductance, Fv/Fm, and electron transport rate (ETR).

### 2.4 Quantitative Real‐Time PCR Analysis

Leaf samples collected in 2025 were immediately frozen in liquid nitrogen and stored at −80°C until RNA extraction. Total RNA was extracted using TRIzol Reagent (Invitrogen, Waltham, MA, USA), according to the manufacturer's instructions. RNA integrity was checked by agarose gel electrophoresis. First‐strand cDNA was synthesized from 1 μg of total RNA using the PrimeScript 1st strand cDNA synthesis kit (Takara, Kyoto, Japan).

Quantitative real‐time PCR (qRT‐PCR) was performed using BlasTaq 2X qPCR MasterMix (Applied Biological Materials, Richmond, BC, Canada) on a real‐time PCR system (CFX Duet real‐time PCR system, Bio‐Rad Laboratories, Hercules, CA, USA). For each treatment history, four biological replicate samples were prepared. Each biological replicate consisted of pooled leaf tissue from three independent plants. Each biological replicate was analyzed in two technical qPCR reactions. Primer sequences used in this study are listed in Table S2. To minimize potential batch effects, plants used in the experiment were propagated from the same cutting cohort, transplanted on the same date, and managed under the same field conditions except for irrigation. Physiological measurements were conducted using the same instruments and protocols within each sampling date. For qRT‐PCR analysis, leaf samples from both treatment histories were collected at the same developmental stage and processed in parallel using the same RNA extraction protocol, cDNA synthesis kit, qPCR master mix, and qPCR instrument. When multiple qPCR plates were required, samples from both treatment histories were included on each plate to avoid confounding treatment history with plate effects.

Relative expression levels were calculated using the 2^−ΔΔCq^ method (Livak and Schmittgen 2001), with *VlActin* used as the internal reference gene. For each target gene, ΔCq values were calculated by subtracting the Cq value of the reference gene from that of the target gene, and ΔΔCq values were calculated relative to the mean value of the irrigated treatment. Relative gene expression levels are presented as fold changes relative to the calibrator sample.

### 2.5 Statistical Analysis

All statistical analyses were performed using IBM SPSS Statistics version 20 (IBM Corp., Armonk, NY, USA). Differences between irrigated and nonirrigated treatments were evaluated separately for each response variable. Final traits measured once at the end of the 2024 irrigation treatment and traits measured once in 2025 were analyzed using one‐way analysis of variance (ANOVA). Seasonal traits measured during the 2024 growing season were analyzed in two complementary ways. To assess temporal response patterns, treatment differences at each sampling date were analyzed using one‐way ANOVA and are presented in Figures S1 and S2. To summarize the overall seasonal effect of irrigation, seasonal mean values were calculated for each trait and used for the main treatment comparisons presented in the main text. Plant height was additionally evaluated at each sampling date to assess temporal growth responses during the season. Data are presented as means ± SE, and differences were considered significant at *p* ≤ 0.05. Because the irrigation treatment was not replicated at the plot level, statistical comparisons based on individual plants were used as exploratory plant‐level contrasts within this field system. These comparisons should not be interpreted as strict inferential tests of replicated plot‐level treatment effects. For qRT‐PCR analysis, technical replicates were averaged, and the four pooled biological replicates per treatment history were used for exploratory comparisons of relative gene expression.

For each trait, the natural‐log response ratio (lnRR) was calculated as

$$\text{lnRR}=\ln\left(\frac{\text{meanI}}{\text{meanN}}\right),$$

where meanI and meanN represent the mean values of the irrigated and nonirrigated plots, respectively. The variance of lnRR was approximated as

$$\mathrm{Var}\left(\text{lnRR}\right)=\left(\frac{{\mathrm{SEI}}^{2}}{{\text{meanI}}^{2}}\right)+\left(\frac{{\mathrm{SEN}}^{2}}{\text{mean}N^{2}}\right),$$

where SEI and SEN are the standard errors of the irrigated and non‐irrigated plots, respectively. The 95% confidence interval was calculated as

$$\text{lnRR}\pm 1.96\times \sqrt{\mathrm{Var}\left(\text{lnRR}\right)}.$$

Confidence intervals were used to summarize the uncertainty of exploratory plant‐level contrasts. Intervals not overlapping zero were interpreted as indicating consistent directional differences, but not as replicated plot‐level treatment inference. The sample sizes used for InRR estimates in forest plots are provided in Table S3.

## 3 Results

### 3.1 Environmental Conditions During Irrigation Treatment

**FIGURE 1:**
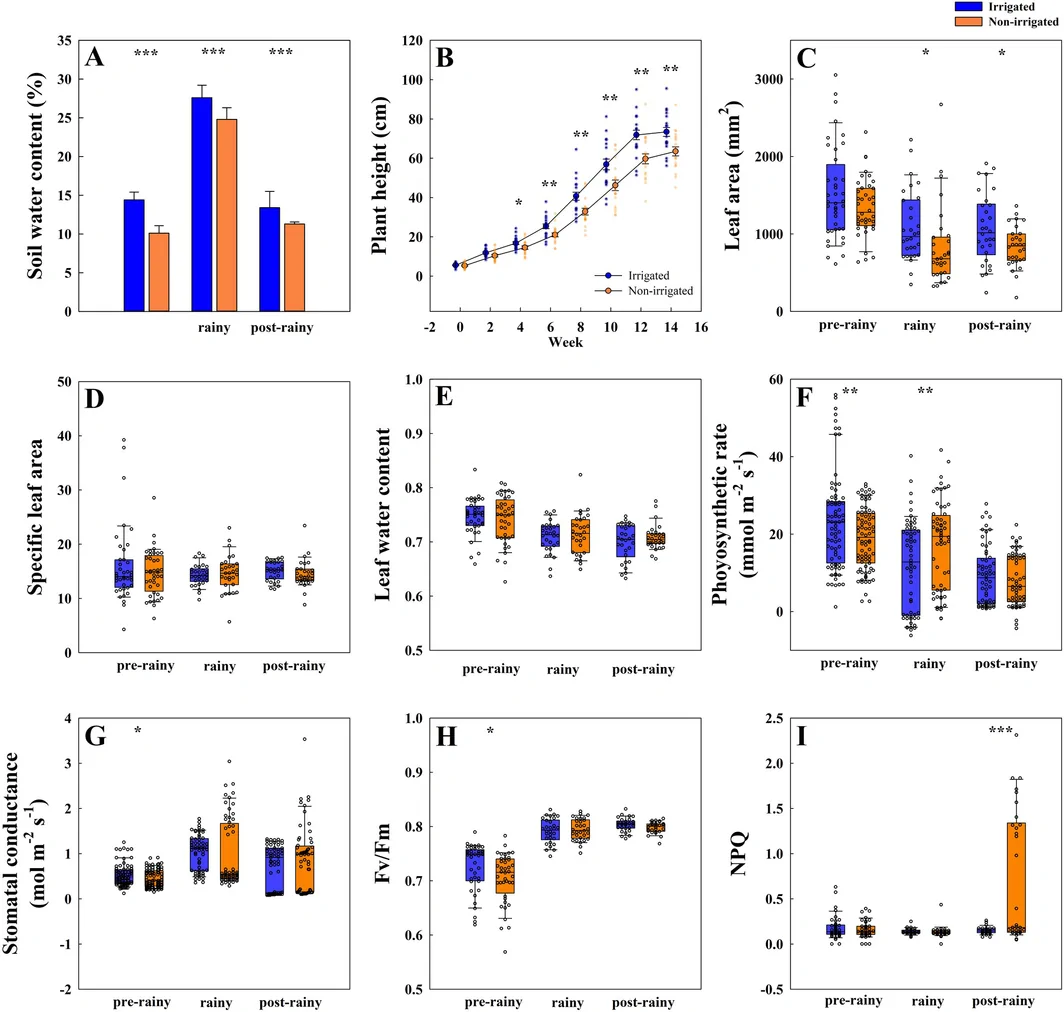
Seasonal soil water content (A) and plant growth responses (B–I) under irrigation treatments. The period was categorized into prerainy (up to week 9), rainy (weeks 10–14), and postrainy (weeks 15–20) phases in 2024. Sample sizes differ among panels: *N* = 20 (per week) for the plant height (B); *n* = 40 (prerainy) and *n* = 30 (rainy and postrainy) for the leaf area (C), specific leaf area (D), leaf water content (E), maximum quantum efficiency of photosystem II (Fv/Fm, H), and non‐photochemical quenching (NPQ, I); and *n* = 80 (prerainy) and *n* = 60 (rainy and postrainy) for the photosynthetic rate (F) and stomatal conductance (G). *, **, and *** indicate significance at *p* < 0.05, 0.01, and 0.001, respectively (analysis of variance).

To assess seasonal environmental effects under climate conditions observed in 2024, environmental variables, including soil water content, were monitored in two plots differing only in irrigation treatment (Table S4). Across all seasons, the irrigated plot consistently exhibited significantly higher soil water content than the non‐irrigated plot (F (1, 278) = 6.709, *p* = 0.010; Figure 1A). Seasonal variation in soil water content was evident. During the rainy period, soil water content exceeded the estimated favorable range in both plots, reaching 27.6% in the irrigated plot and 24.8% in the nonirrigated plot. Thus, although drought stress was unlikely during this period, both plots experienced temporarily excessive soil moisture, with the irrigated plot showing the higher value. In contrast, during the prerainy and postrainy periods, soil water content averaged 14.4% and 13.4% in the irrigated plot and 10.1% and 11.3% in the nonirrigated plot, respectively. Particle‐size analysis showed that the experimental soil consisted of 84.13% sand, 12.63% silt, and 3.24% clay. Based on this soil texture, the favorable volumetric soil water content for plant growth was estimated to range from approximately 8%–18% (Jones 2007; Brady and Weil 2008; Taiz et al. 2015). Therefore, soil water content in both plots remained within or above the estimated favorable range throughout the treatment period, suggesting that severe drought stress was unlikely even in the nonirrigated plot.

### 3.2 Seasonal Plant Growth Response During Irrigation Treatment

Seasonal plant growth responses during irrigation treatment in 2024 were investigated every two weeks. Plant height began to differ significantly from week 4, with the difference expanding to 9.9 cm by week 14 (Figure 1B), at which point flowering occurred and subsequent stem elongation no longer progressed. Leaf area did not differ between treatments during the prerainy period. However, during the rainy and postrainy periods, leaf area was higher in the irrigated plot (rainy: F (1,58) = 4.214, *p* = 0.045; postrainy: F (1,58) = 6.373, *p* = 0.014), corresponding to increases of 31.1% and 28.7%, respectively (Figure 1C; Figure S1A). Seasonal SLA and leaf water content did not differ significantly between treatments (Figure 1D,E; Figure S1B,C). Significant differences in photosynthetic rate were observed during the prerainy (F (1,158) = 7.105, *p* = 0.008) and rainy periods (F (1,118) = 7.065, *p* = 0.009), whereas stomatal conductance differed significantly only during the prerainy period (F (1,158) = 4.920, *p* = 0.028; Figure 1F,G; Figure S2A,B). During the prerainy period, photosynthetic rate was higher in the irrigated plot, reaching 23.3 μmol·m^−2^·s^−1^, compared with 21.0 μmol·m^−2^·s^−1^ in the nonirrigated plot. In contrast, during the rainy period, photosynthetic rate was greater in the nonirrigated plot, with a mean value of 17.3 μmol·m^−2^·s^−1^, than in the irrigated plot, which averaged 11.7 μmol·m^−2^·s^−1^. During the rainy and postrainy periods, Fv/Fm in both plots exceeded 0.78. During the prerainy period, Fv/Fm differed between treatments, with values of 0.73 in the irrigated plot and 0.70 in the nonirrigated plot (F (1,78) = 4.419, *p* = 0.039; Figure 1H; Figure S2C). NPQ remained below 0.2 in both plots during the prerainy and rainy periods, with no significant differences observed between the irrigated and nonirrigated plots (Figure 1I; Figure S2D). In the postrainy period, NPQ in the irrigated plot remained low (0.15), showing little deviation from values observed in the earlier periods. In contrast, NPQ in the nonirrigated plot increased sharply to 0.74, resulting in a significant difference between treatments (F (1,58) = 18.706, *p* < 0.001).

### 3.3 Final Growth and Reproductive Responses at the End of the Irrigation Treatment

**FIGURE 2:**
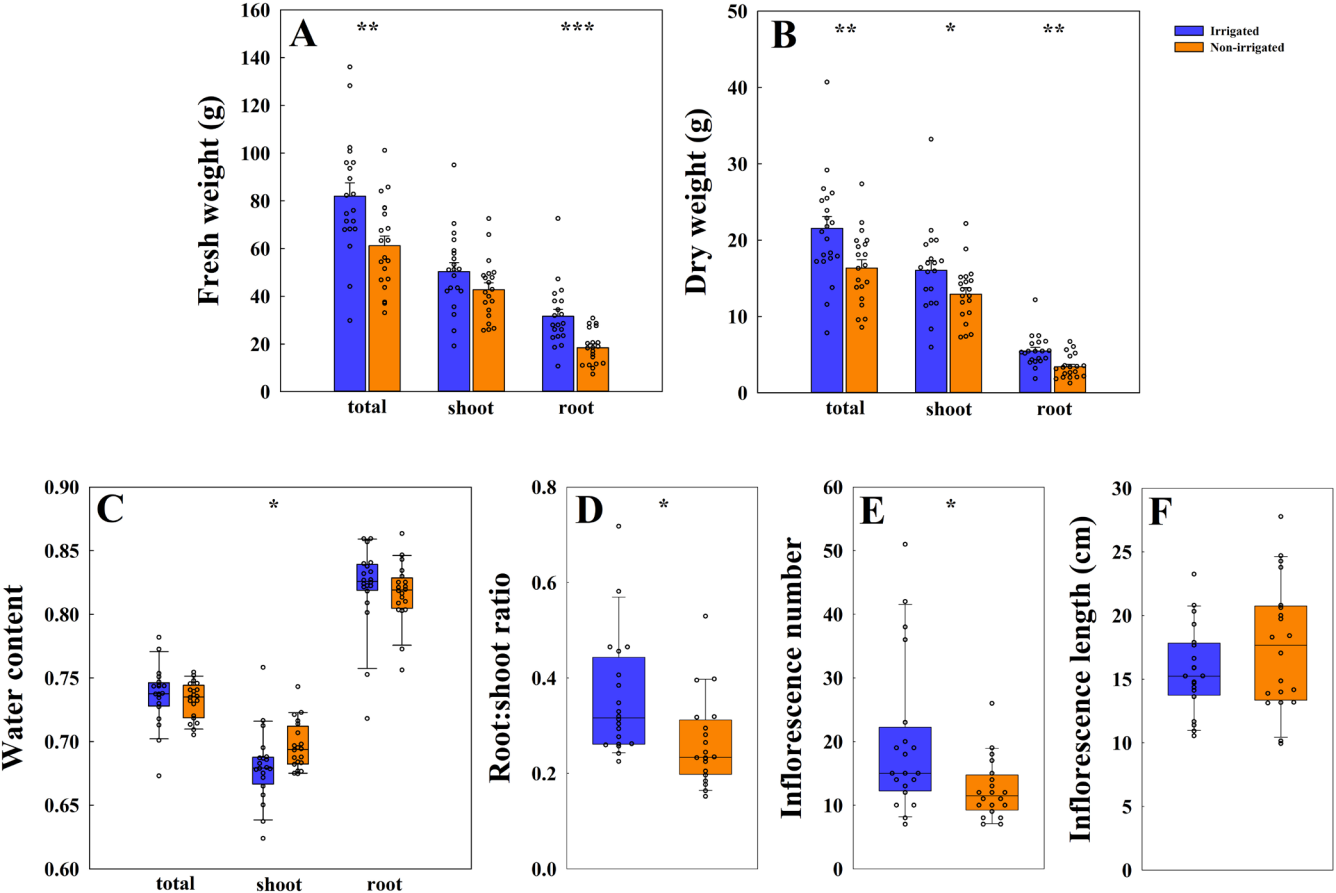
Plant growth responses after irrigation treatment period in 2024. *, **, and *** indicate significance at *p* < 0.05, 0.01, and 0.001, respectively (analysis of variance; *n* = 20).

At the end of the 2024 irrigation treatment, above‐ground fresh weight did not differ significantly between treatments, whereas significant positive responses to irrigation were shown in total fresh weight (F (1,38) = 8.980, *p* = 0.005), root fresh weight (F (1,38) = 15.482, *p* < 0.001), total dry weight (F (1,38) = 7.747, *p* = 0.008), shoot dry weight (F (1,38) = 4.211, *p* = 0.047) and root dry weight (F (1,38) = 12.971, *p* = 0.001; Figure 2A,B). There was no significant difference between treatments in total water content and root water content. However, a significant difference was detected in shoot water content (F (1,38) = 4.359, *p* = 0.044; Figure 2C). The root: shoot ratio (RSR), calculated based on aboveground and belowground dry weights, was significantly higher in irrigated plots (F (1,38) = 7.280, *p* = 0.010; Figure 2D).

Reproductive traits also differed between treatments. The number of inflorescences was significantly greater in irrigated plants (F (1,38) = 6.610, *p* = 0.014, Figure 2E), whereas inflorescence length did not differ significantly between treatments (Figure 2F). In contrast, significant differences were observed in seed length (F (1,18) = 6.683, *p* = 0.019), seed width (F (1,18) = 4.425, *p* = 0.050), and seed mass (F (1,8) = 30.851, *p* = 0.001; Table 1). Seeds produced by irrigated plants exhibited increases of approximately 16.7% in seed mass, 14.4% in seed length, and 14.2% in seed width compared with those from nonirrigated plants. The forest plot of lnRR showed positive effect sizes for most biomass‐ and reproduction‐related traits, whereas water‐content‐related traits were clustered near zero (Figure 5A).

**TABLE 1:** Seed traits of *Veronica longifolia* , including 100‐seed weight, seed length, and seed width, measured under irrigated and non‐irrigated treatments in 2024.

	100‐seed weight (mg)	Seed length (mm)	Seed width (mm)
Irrigated	10.36 ± 0.17^a^	1.082 ± 0.0360	0.786 ± 0.0354
Non‐irrigated	8.88 ± 0.14	0.946 ± 0.0386	0.688 ± 0.0302
Significance	**	**	*

^a^ Values indicate standard error.

* *p* ≤ 0.05.

** Indicate significance at *p* ≤ 0.01 by analysis of variance (*n* = 5 for seed weight; *n* = 10 for seed length and seed width).

### 3.4 Potential Legacy Effects Associated With Prior‐Year Irrigation History

As described above, the 20‐week irrigation treatment applied in 2024 did not induce pronounced differences in seasonal plant responses, but its cumulative effects significantly influenced biomass accumulation and reproductive performance. To evaluate whether these treatment effects persisted into the following year, no irrigation was applied to either plot in 2025, and plant responses were assessed in March–April and August. In addition, the relative expression levels of selected genes were analyzed.

**FIGURE 3:**
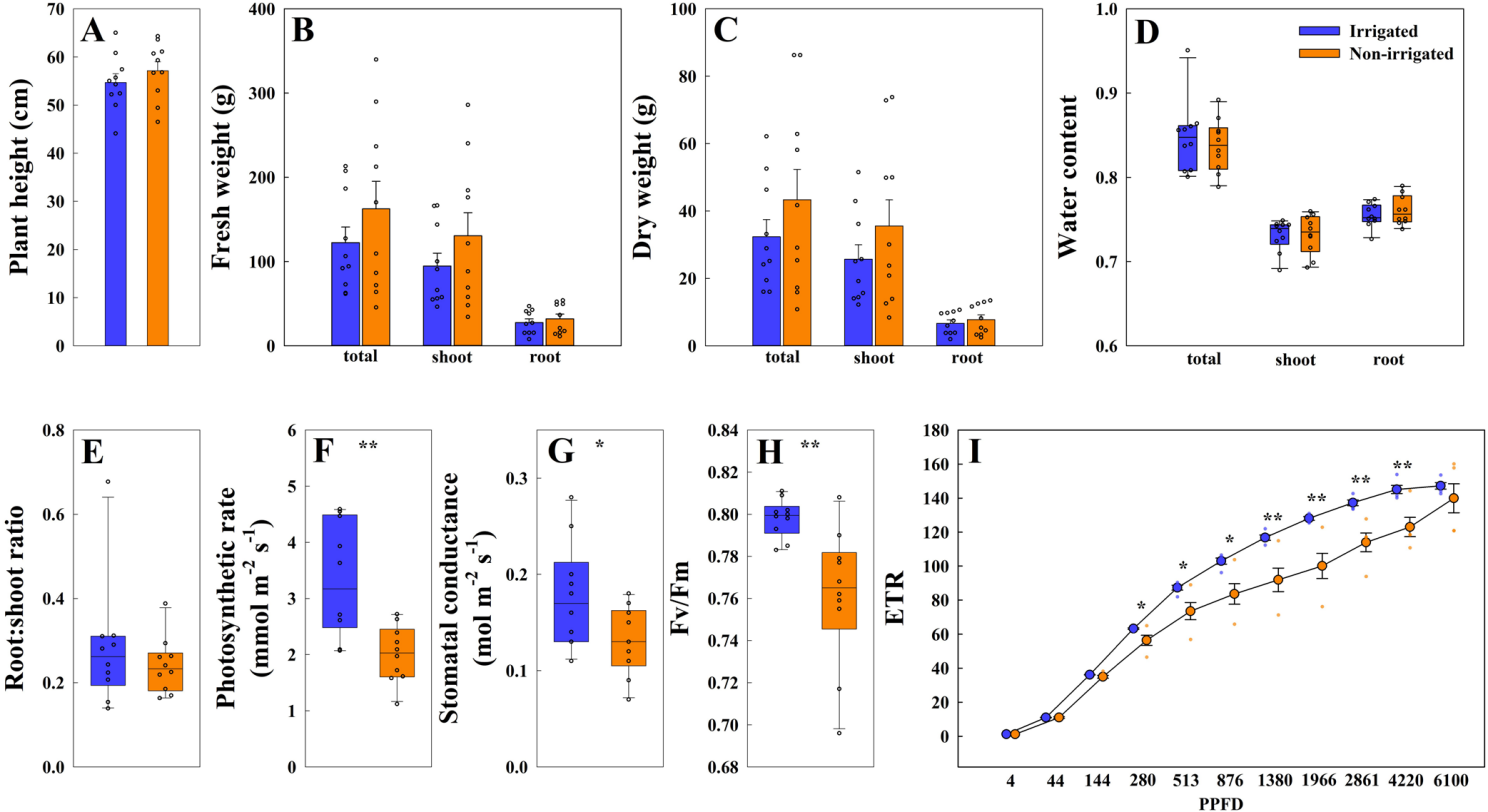
Plant growth responses in August 2025 when neither group was irrigated. Sample sizes differ among panels: *N* = 10 for the plant height (A), fresh weight (B), dry weight (C), water content (D), root: shoot ratio (E), photosynthetic rate (F), stomatal conductance (G), and maximum quantum efficiency of photosystem II (Fv/Fm, H); and *n* = 5 (per photosynthetic photon flux density) for electron transport rate (ETR, I). * and ** indicate significance at *p* < 0.05 and 0.01, respectively (analysis of variance).

In March, no significant difference was observed in the timing of spring shoot emergence between treatment histories, although mean emergence was 0.5 days earlier in plants previously subjected to irrigation. At this stage, previously irrigated plants showed significantly higher photosynthetic rate (F (1,18) = 15.146, *p* = 0.001) and stomatal conductance (F (1,18) = 6.680, *p* = 0.019; Table 2). In August, a broader range of traits was examined. No significant differences were observed between treatments in plant height, fresh weight, or dry weight (Figure 3A–C). In contrast to the patterns observed in 2024, these traits tended to be higher in plants previously subjected to the nonirrigated plot. Similarly, tissue water content and the RSR did not differ significantly between treatments (Figure 3D,E). However, plants previously exposed to irrigation had higher photosynthetic rate (F (1,18) = 13.812, *p* = 0.002), stomatal conductance (F (1,18) = 4.972, *p* = 0.039), Fv/Fm (F (1,18) = 11.602, *p* = 0.003; Figure 3F–H) than plants previously exposed to the nonirrigated treatment. ETR was also higher in previously irrigated plants at photosynthetic photon flux densities of 280, 513, 876, 1380, 1966, 2861, and 4220 μmol·m^−2^·s^−1^ (F (1,8) = 5.355, 6.807, 9.482, 12.256, 14.055, 16.168, and 12.847, respectively; *p* = 0.049, 0.031, 0.015, 0.008, 0.006, 0.004, and 0.007, respectively; Figure 3I).

**TABLE 2:** Legacy effects of prior irrigation on shoot appearance, photosynthetic rate, and stomatal conductance of *Veronica longifolia* measured in 2025 under nonirrigated conditions.

	Time taken for shoot appearance (day)	Photosynthetic rate (μmol·m^−2^·s^−1^)	Stomatal conductance (mol·m^−2^·s^−1^)
Irrigated	2.5 ± 0.25^a^	5.94 ± 0.177	0.21 ± 0.008
Nonirrigated	3.0 ± 0.48	5.01 ± 0.198	0.18 ± 0.010
Significance	ns	^**^	^**^

^a^ Values indicate standard errors.

** Indicate no significance and significance at *p* ≤ 0.01 by analysis of variance (*n* = 34 for time taken for shoot appearance; *n* = 10 for photosynthetic rate and stomatal conductance).

A forest plot summarizing the effects of prior‐year irrigation history on growth and physiological traits in 2025 showed that most biomass‐related traits were centered near zero or had confidence intervals overlapping zero, whereas physiological traits were shifted in the positive direction (Figure 5B). The effect size for the time taken for shoot appearance was slightly negative, but the corresponding comparison was not significant.

**FIGURE 4:**
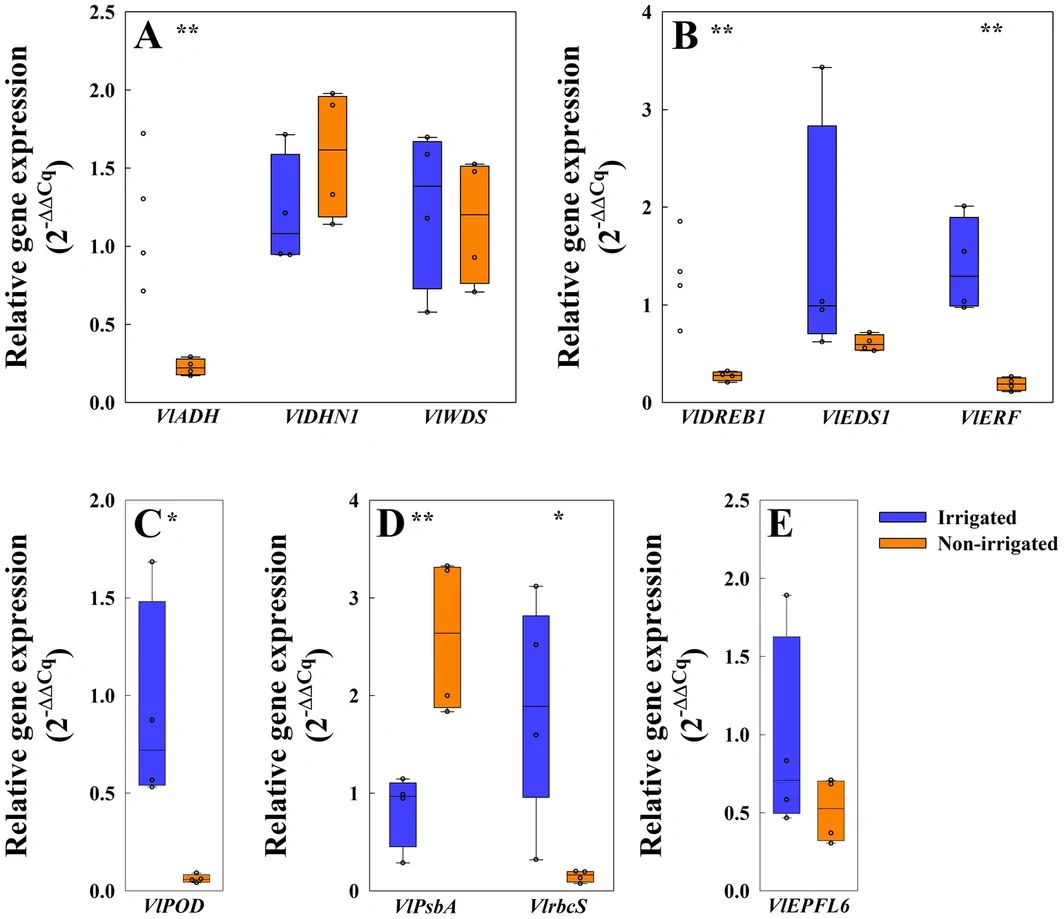
Relative expression of selected genes in *Veronica longifolia* in 2025 following prior irrigated and non‐irrigated treatments. Relative transcript abundance was normalized to *VlActin* and calculated using the 2^−ΔΔCq^ method. Bars represent means ± SE of four biological replicates. Each biological replicate consisted of pooled leaf tissue from three independent plants. * and ** indicate significance at *p* < 0.05 and 0.01, respectively (analysis of variance).

To examine whether prior irrigation history was associated with differences in transcript abundance in the following year, the expression levels of ten selected genes commonly associated with abiotic stress responses, transcriptional regulation, antioxidant defense, photosynthesis, and stomatal development were analyzed in August 2025. Among the selected genes, *VlADH* showed higher expression in plants previously subjected to irrigation (irrigated = 1.173; nonirrigated = 0.227; F (1,6) = 18.426, *p* = 0.005; Figure 4A). *VlDREB1* and *VlERF* also showed higher expression in previously irrigated plants (*VlDREB1*: irrigate*d* = 1.281; nonirrigated = 0.272; F (1,6) = 18.834, *p* = 0.005; *VlERF*: irrigated = 1.392; nonirrigated = 0.189; F (1,6) = 24.115, *p* = 0.003; Figure 4B). *VlPOD* expression was also higher in previously irrigated plants (irrigated = 0.914; nonirrigated = 0.062; F (1,6) = 10.075, *p* = 0.019; Figure 4C). Among photosynthesis‐related genes, *VlpsbA* showed higher expression in plants previously subjected to the non‐irrigated treatment (irrigated = 0.840; nonirrigated = 2.609; F (1,6) = 15.832, *p* = 0.007), whereas *VlrbcS* showed higher expression in previously irrigated plants (irrigated = 1.887; nonirrigated = 0.150; F (1,6) = 8.117, *p* = 0.029; Figure 4D). *VlEPFL6* did not differ between treatments (Figure 4E).

**FIGURE 5:**
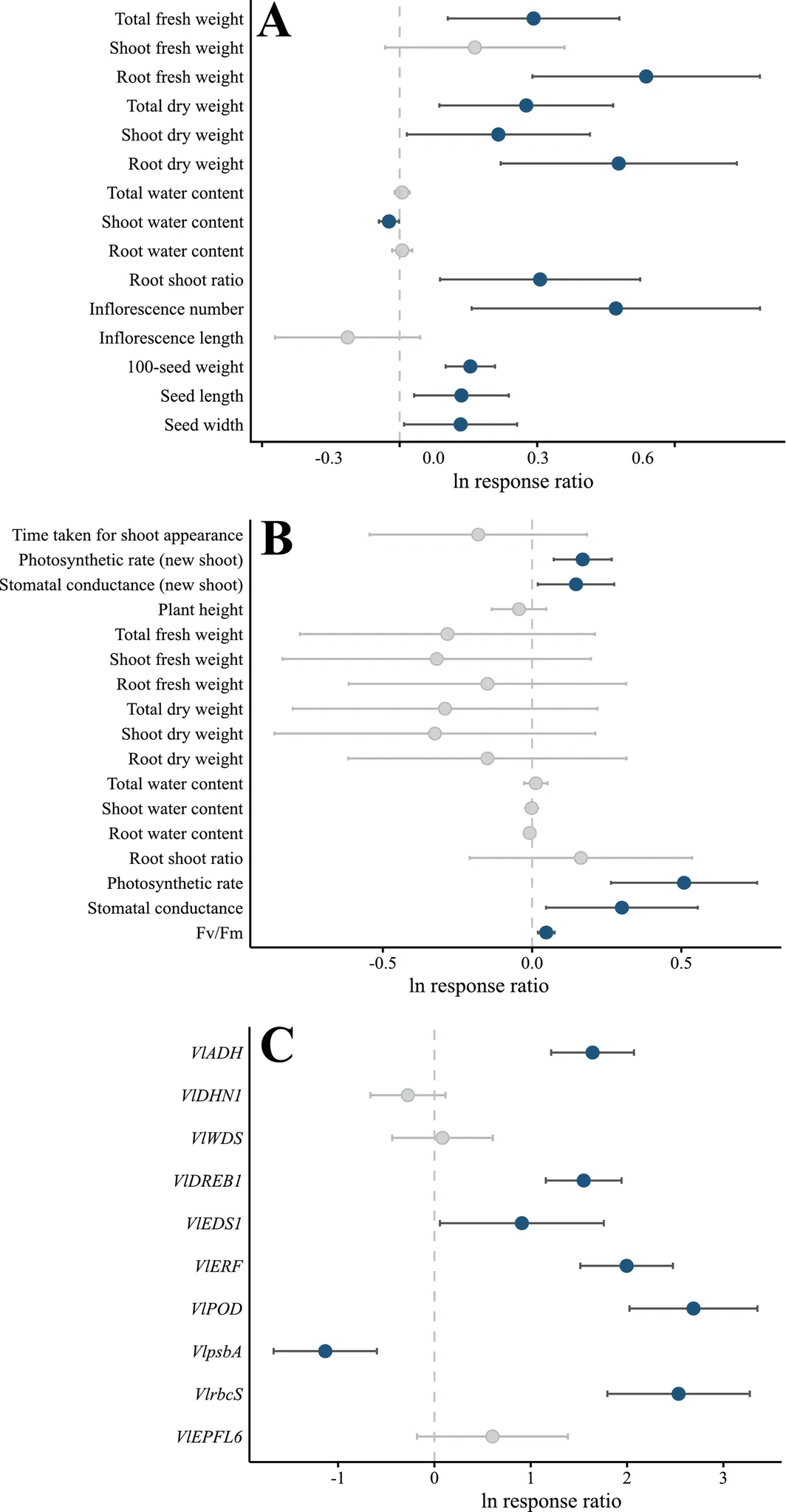
Forest plots of natural‐log response ratios [ln (irrigated/nonirrigated)] summarizing irrigation‐associated plant‐level contrasts for final growth and reproductive traits in 2024 (A), growth and physiological traits in 2025 (B), and relative gene expression in 2025 (C). Horizontal bars indicate 95% confidence intervals. Positive values indicate higher values in irrigated or previously irrigated plants, whereas negative values indicate higher values in nonirrigated or previously non‐irrigated plants. Confidence intervals not overlapping zero indicate consistent directional differences in exploratory plant‐level contrasts, but should not be interpreted as replicated plot‐level treatment inference. Trait‐specific sample sizes are provided in Table S3.

The forest plot of relative gene expression summarized these treatment‐history‐associated differences (Figure 5C). Positive effect sizes were observed for *VlADH*, *VlDREB1*, *VlERF*, *VlPOD*, and *VlrbcS*, whereas *VlpsbA* showed a negative effect size, indicating relatively higher expression in plants previously subjected to the nonirrigated treatment. These results indicate that prior irrigation history was associated with selective differences in transcript abundance, but they do not by themselves demonstrate a specific physiological memory mechanism.

## 4 Discussion

This study was conducted as a baseline investigation to anticipate the impacts of future climate‐change–driven extreme climatic events on plants in the Korean Peninsula by examining irrigation‐dependent responses of *V. longifolia* under current field conditions. Throughout the experimental period, soil water content in both the irrigated and nonirrigated plots remained within or above the estimated favorable range for plant growth, indicating that the 2024 irrigation treatment did not create a contrast between severe drought and normal water availability. This interpretation is also consistent with national climatic conditions in 2024, when the number of meteorological drought days recorded in Korea was the lowest since 1993 (KMA 2025). Therefore, the growth and physiological differences observed in this study should be interpreted as responses to moderate variation in relative field water availability rather than as responses to severe drought stress.

Nevertheless, the rainy period presented a different moisture context. Soil water content exceeded the estimated favorable range in both plots and approached a near‐saturated condition, particularly in the irrigated plot. This temporary excess moisture may have contributed to the lower photosynthetic rate observed in the irrigated plot during the rainy period, possibly through mild waterlogging‐related constraints on root‐zone aeration. Because the soil moisture values used here represent seasonal means, however, they may not fully capture short‐term extreme events such as brief droughts, heavy rainfall, or transient flooding. Thus, the climatic conditions of 2024 should not be considered fully representative of future climate‐change scenarios, but rather as a field baseline for evaluating how *V. longifolia* responds to moderate fluctuations in water availability.

Seasonal plant growth responses showed enhanced performance in the irrigated treatment, where soil moisture availability was relatively higher, as indicated by increased plant height. Leaf area was also greater in the irrigated treatment; however, the absence of significant differences in leaf water content and SLA suggests that this increase was not a stress‐induced response but rather reflected enhanced growth under sufficient water availability. The lack of significant differences in Fv/Fm further indicates that growth inhibition due to drought stress did not occur in the nonirrigated treatment (Maxwell and Johnson 2000). The low Fv/Fm in the prerainy period is unlikely to be directly attributable to soil moisture conditions. Rather, it may have resulted from relatively high irradiance during the early growth stage or complex with other environmental cues (Table S4). Interestingly, photosynthetic rates exhibited a reversal between the prerainy and rainy periods. This pattern may be related to rainy‐period excess soil moisture, particularly in the irrigated plot, which could have constrained root‐zone aeration and suppressed photosynthesis (Yan et al. 2018). During the postrainy period, NPQ measurements were conducted on three occasions, and a marked increase in NPQ was observed in the nonirrigated treatment during late August and early September. During these periods, mean NPQ values reached 0.98 and 1.11, respectively. Given that Fv/Fm values remained within the normal range and photosynthetic rates did not differ significantly between treatments, these elevated NPQ values indicate enhanced photoprotective activity rather than photosynthetic inhibition (Maxwell and Johnson 2000; Baker 2008). This response likely reflects a regulatory adjustment to increased irradiance and temperature accompanied by declining soil moisture following the rainy period.

Differences in soil water content induced by the irrigation treatment led to cumulative effects on plant growth, resulting in significant differences in biomass between the two treatments. In particular, the irrigated plants exhibited a higher proportion of belowground biomass, which contrasts with previous studies reporting increased root growth under water‐deficient conditions (Dao et al. 2025). However, because soil water content in the present study did not decline to levels that impose drought stress, the observed differences in RSR are more reasonably interpreted as a consequence of differences in growth conditions rather than as an adaptive response to dry soil. When soil moisture is stably maintained, maize and lettuce tend to exhibit enhanced shoot growth, leading to a reduced RSR (Zhang, Ji, et al. 2022; Zhang, Wang, et al. 2022; Liu et al. 2025). In contrast, *V. longifolia* ceases shoot elongation after flowering and continues to produce inflorescences, a growth characteristic that may limit carbon allocation to vegetative aboveground tissues. Consequently, in the irrigated treatment, where overall growth was more vigorous, continued accumulation of root biomass likely contributed to the higher proportion of belowground biomass observed. Meanwhile, the cumulative effects of irrigation also influenced the reproductive performance of *V. longifolia* . Plants in the irrigated plot produced more inflorescences, and their seeds exhibited greater size and mass compared with those of plants in the nonirrigated plot. Consistent with the growth responses described above, these differences in reproductive traits are likely attributable to the overall enhanced vegetative growth under irrigated conditions, which increased carbon availability for reproductive development. The effect‐size summary shown in Figure 5A reinforces this interpretation by demonstrating that irrigation effects were generally positive across biomass and reproductive traits, while responses in tissue water content remained comparatively small. This pattern indicates that under the relatively mild moisture contrast observed in 2024, irrigation did not simply improve plant water status at the time of harvest, but rather promoted cumulative gains in growth and reproductive performance over the course of the season.

In contrast to 2024, when irrigation treatments were applied, no significant differences in biomass were observed between the two treatment groups in 2025, during which neither group received irrigation. This result supports the interpretation that the biomass differences observed in 2024 were a relative outcome driven by differences in soil water availability. Interestingly, although not statistically significant, plants from the nonirrigated treatment tended to exhibit greater plant height and biomass in 2025, whereas photosynthetic rate, stomatal conductance, Fv/Fm, and ETR were consistently higher in plants previously subjected to irrigation. This suggests that, despite similar biomass accumulation, plants in the irrigated treatment maintained a relatively higher level of physiological activity.

Notably, plants in the irrigated treatment experienced a marked shift in soil moisture conditions, transitioning from persistently high soil water availability during the 2024 irrigation period to a completely nonirrigated condition in 2025. To investigate potential molecular responses associated with this environmental transition, relative gene expression levels were analyzed using qRT‐PCR. *ADH* is a well‐established marker gene for hypoxic and waterlogging stress, with expression typically induced under low‐oxygen conditions (Dennis et al. 2000; Bailey‐Serres and Voesenek 2008). However, the elevated expression of *VlADH* observed in leaves sampled in August 2025 cannot be directly attributed to waterlogging stress, because excess soil moisture was not confirmed at the time of sampling. Given that gene expression was measured at a single time point, this pattern should not be interpreted as direct evidence of stress memory or priming. Rather, the higher *VlADH* expression in previously irrigated plants indicates a treatment‐history‐associated transcriptional difference. One possible explanation is that prior exposure to higher soil moisture altered subsequent metabolic or oxygen‐related regulatory status (Kreuzwieser and Rennenberg 2014), but additional time‐course measurements would be required to determine whether this pattern represents a memory‐like response.

*DREB1* is a key transcription factor commonly upregulated in response to abiotic stresses such as drought, low temperature, and salinity (Feng et al. 2019; Zhou et al. 2019). However, recent studies have demonstrated that *DREB1* can also be involved in metabolic regulation, including the accumulation of soluble sugars, and may be upregulated under physiologically active conditions that do not necessarily involve severe stress (Zhao et al. 2023; Zhang and Xia 2023). In this study, the increased expression of *VlDREB1* in the irrigated treatment was consistent with the higher Fv/Fm, photosynthetic rate, stomatal conductance, and ETR observed in these plants. Therefore, rather than indicating an active drought‐stress response, the elevated expression of *VlDREB1* may reflect a treatment‐history‐associated transcriptional pattern linked to the higher physiological activity observed in previously irrigated plants in August 2025.

*ERF* transcription factors are involved in ethylene signaling and can participate in both stress‐related and developmental regulation, including responses to abiotic and biotic stimuli, leaf development, stomatal regulation, and photosynthetic performance (Licausi et al. 2013; Müller and Munné‐Bosch 2015; Dubois et al. 2018; Xie et al. 2019; Sanchez‐Munoz et al. 2025). In this study, *VlERF* expression was higher in plants previously subjected to irrigation. Because no severe drought stress was evident during the treatment year and gene expression was measured only once in the following year, this result should not be interpreted as evidence of a defined ethylene‐mediated stress‐memory mechanism. Instead, it indicates that prior irrigation history was associated with altered expression of an ethylene‐related regulatory gene, which may be linked to differences in physiological status between the two plant groups.

Peroxidase (*POD*) plays a crucial role in mitigating oxidative stress by scavenging excessive ROS, thereby protecting photosynthetic tissues and maintaining a balance between growth and defense responses (Li et al. 2024). The higher photosynthetic activity observed in the irrigated treatment implies enhanced photosynthetic electron transport and metabolic fluxes, which are likely accompanied by increased intracellular ROS production. The higher expression of *VlPOD* in previously irrigated plants may indicate a difference in antioxidant‐related transcriptional status between treatment histories. However, because ROS levels and antioxidant enzyme activities were not measured in 2025, this result should not be taken as direct evidence that previously irrigated plants had enhanced antioxidant buffering capacity. Instead, *VlPOD* expression provides a potential molecular indicator that could be examined in future studies together with direct measurements of oxidative status.

In contrast, *psbA* encodes the D1 protein of the PSII reaction center and is upregulated in response to photodamage or imbalances in electron transport to facilitate PSII repair (Tikkanen and Aro 2014). Meanwhile, *rbcS* regulates carbon fixation capacity and photosynthetic carbon assimilation, and its upregulation reflects enhanced photosynthetic performance (Spreitzer and Salvucci 2002). Taken together, plants in the irrigated treatment maintained active photosynthetic electron transport and carbon fixation, accompanied by a strengthened antioxidant system to control ROS generation. The opposite expression patterns of *VlpsbA* and *VlrbcS* may reflect different photosynthesis‐related transcriptional states between the two treatment histories. Higher *VlrbcS* expression in previously irrigated plants was consistent with their higher photosynthetic activity, whereas higher *VlpsbA* expression in previously nonirrigated plants may indicate a greater requirement for PSII repair‐related transcript accumulation. However, because PSII repair dynamics were not directly measured, this interpretation remains hypothetical.

The integrated effect‐size patterns in Figure 5B,C indicate that prior irrigation history was associated more strongly with physiological and selected transcriptional traits than with biomass accumulation in 2025. While biomass differences largely disappeared under uniform nonirrigated conditions, plants previously subjected to irrigation maintained higher photosynthetic performance and showed selective differences in transcript abundance. These patterns suggest possible carry‐over physiological and transcriptional differences, although they do not demonstrate a specific stress‐memory mechanism.

Overall, our results suggest that, under the field conditions observed in this study, persistent differences in soil water availability were associated with differences in growth, physiology, and selected transcript abundances in *V. longifolia* , even though severe drought stress was unlikely. These findings highlight the value of integrating physiological measurements with targeted gene expression analyses, while also emphasizing the need for replicated field experiments and time‐course molecular sampling to determine whether such patterns represent true physiological memory or other carry‐over effects of prior growth conditions.

A major limitation of this study is that the irrigation treatment was implemented at the plot level, with one irrigated plot and one nonirrigated plot. Thus, individual plants within each plot were plant‐level subsamples rather than true independent replicates of the irrigation treatment. As a result, the observed differences should not be interpreted as definitive irrigation treatment effects in a strict inferential sense, but rather as biologically informative plot‐level contrasts within the present field system. Nevertheless, repeated measurements across seasons and the consistent patterns observed across growth, physiological, and transcriptional traits support the biological relevance of the cumulative and potential carry‐over responses identified in this study.

## Funding

This work was supported by the Korea National Arboretum of the Korea Forest Service (Development of Breeding Models for Native Garden Plants in the New Climate Regime, KNA 1‐5‐1‐24‐1).

## Conflicts of Interest

The authors declare no conflicts of interest.

## Supporting information


**Figure S1:** Weekly leaf responses under irrigation treatments for 20 weeks. Seasons are categorized into prerainy (up to week 9), rainy (week 10–14), and postrainy (week 15–20). * and ** indicate significance at *p* < 0.05 and 0.01, respectively (ANOVA; *n* = 10).
**Figure S2:** Weekly photosynthetic (A, B) and physiological (C, D) responses under irrigation treatments for 20 weeks. Seasons are categorized into prerainy (up to week 9), rainy (week 10–14), and postrainy (week 15–20). *, ** and *** indicate significance at p < 0.05, 0.01, and 0.001, respectively (ANOVA; n = 20 for A, B; n = 10 for C, D).
**Table S1:** Plant growth, physiological, and genetic response parameters measured and measurement methods used in this study.
**Table S2:** Gene information and primers used for quantitative reverse transcript PCR (qRT‐PCR) analysis.
**Table S3:** Sample sizes for ln response ratio estimates in forest plots.
**Table S4:** Monitored seasonal environmental conditions during irrigation treatment.

## Data Availability

The data that support the findings of this study are openly available in Zenodo (Kim et al. 2026) at https://doi.org/10.5281/zenodo.21467416.
