# Supplementary material for: Cumulative and Legacy Effects of Irrigation on Growth, Physiology, and Gene Expression in Veronica longifolia Under Field Conditions
Source: Plant Environ Interact. 2026 Sep 3;7(5):e70202. doi: 10.1002/pei3.70202 (PMC13539903; doi:10.1002/pei3.70202)
Supplement: Supplementary file 1 [file PEI3-7-e70202-s001.docx]

**Supplementary materials**

**Supplementary figure S1.** Weekly leaf responses under irrigation treatments for 20 weeks. Seasons are categorized into pre-rainy (up to week 9), rainy (week 10-14), and post-rainy (week 15-20). * and ** indicate significance at *P* < 0.05 and 0.01, respectively (ANOVA; n=10).

**Supplementary figure S2.** Weekly photosynthetic (**A, B**) and physiological (**C, D**) responses under irrigation treatments for 20 weeks. Seasons are categorized into pre-rainy (up to week 9), rainy (week 10-14), and post-rainy (week 15-20). *, ** and *** indicate significance at *P* < 0.05, 0.01, and 0.001, respectively (ANOVA; n=20 for **A**, **B**; n=10 for **C**, **D**).

**Supplementary table S1.** Plant growth, physiological, and genetic response parameters measured and measurement methods used in this study.

|  | **Measured variable** | **Definition / Measurement method** | **Plant part** | **Timing** | **Tools** |
| --- | --- | --- | --- | --- | --- |
| **Seasonal growth responses**  **(during irrigation treatments)** | Plant height | Distance from lowest node above ground to the most distal node | Whole plant | Biweekly (2024) |  |
|  | Leaf area | Leaf area measurement | 2^nd^–4^th^ fully expanded leaf from top | Biweekly (2024) | ImageJ v1.54 (NIH, MA, USA) |
|  | Leaf fresh weight |  | 2^nd^–4^th^ fully expanded leaf from top | Biweekly (2024) | Analytical balance |
|  | Leaf dry weight |  | 2^nd^–4^th^ fully expanded leaf from top | Biweekly (2024) | Dry oven (OF-02G Forced convention oven, Jeio Tech, Daejeon, Korea), Analytical balance |
|  | Specific leaf area (SLA) | Leaf area / leaf dry weight | 2^nd^–4^th^ fully expanded leaf from top | Biweekly (2024) |  |
|  | Leaf water content | (leaf fresh weight − leaf dry weight) / leaf fresh weight | 2^nd^–4^th^ fully expanded leaf from top | Biweekly (2024) |  |
|  | Photosynthetic rate |  | 2^nd^–4^th^ fully expanded leaf from top | Biweekly (2024) | Portable photosynthesis system (LCpro, ADC Bioscientific Inc., Hertfordshire, UK) |
|  | Stomatal conductance |  | 2^nd^–4^th^ fully expanded leaf from top | Biweekly (2024) | Portable photosynthesis system |
|  | Fv/Fm | Maximum quantum efficiency of PSII | 2^nd^–4^th^ fully expanded leaf from top | Biweekly (2024) | Fluorometer (PAM-2500, Walz, Bavaria, Germany) |
|  | NPQ | Non-photochemical quenching | 2^nd^–4^th^ fully expanded leaf from top | Biweekly (2024) | Fluorometer |
| **Biomass responses (right after irrigation treatments)** | Total fresh weight |  | Whole plant | After irrigation ends (2024) | Analytical balance |
|  | Total dry weight |  | Whole plant | After irrigation ends (2024) | Dry oven, Analytical balance |
|  | Shoot fresh weight |  | Separated at lowest node | After irrigation ends (2024) | Analytical balance |
|  | Shoot dry weight |  | Separated at lowest node | After irrigation ends (2024) | Dry oven, Analytical balance |
|  | Root fresh weight |  | Separated at lowest node | After irrigation ends (2024) | Analytical balance |
|  | Root dry weight |  | Separated at lowest node | After irrigation ends (2024) | Dry oven, Analytical balance |
|  | Total water content | (Total fresh weight − total dry weight) / total fresh weight | Whole plant | After irrigation ends (2024) |  |
|  | Shoot water content | (Shoot fresh weight − shoot dry weight) / shoot fresh weight | Separated at lowest node | After irrigation ends (2024) |  |
|  | Root water content | (Root fresh weight − root dry weight) / root fresh weight | Separated at lowest node | After irrigation ends (2024) |  |
|  | Root:shoot ratio | Root dry weight/shoot dry weight | Same as above | After irrigation ends (2024) |  |
|  | Inflorescence length (mean) | Total inflorescence length / number of inflorescences | All inflorescences on each plant | After irrigation ends (2024) |  |
|  | Number of inflorescences |  | Whole plant | After irrigation ends (2024) |  |
|  | Seed length | Length of 20 randomly selected seeds | Seeds | After irrigation ends (2024) | Calipers |
|  | Seed width | Width of 20 randomly selected seeds | Seeds | After irrigation ends (2024) | Calipers |
|  | Seed weight | 100-seed weight | Seeds | After irrigation ends (2024) | Analytical balance |
| **Legacy responses (2025)** | Shoot emergence date | Date when ≥2 true leaves fully expanded |  | March 2025 |  |
|  | Photosynthetic rate |  | 2025 new shoots | April & August 2025 |  |
|  | Stomatal conductance |  | 2025 new shoots | April & August 2025 |  |
|  | Total fresh and dry weight |  | Same as 2024 | August 2025 | Dry oven, Analytical balance |
|  | Shoot fresh and dry weight |  | Same as 2024 | August 2025 | Dry oven, Analytical balance |
|  | Root fresh and dry weight |  | Same as 2024 | August 2025 | Dry oven, Analytical balance |
|  | Total, shoot, and root water content | Same as 2024 | Same as 2024 | August 2025 |  |
|  | Root:shoot ratio | Same as 2024 | Same as 2024 | August 2025 |  |
|  | Fv/Fm | Same as 2024 | Same as 2024 | August 2025 |  |
|  | ETR | Electron transport rate | 2^nd^–4^th^ fully expanded leaf from top | August 2025 | Fluorometer |

**Supplementary table S2.** Gene information and primers used for quantitative reverse transcript PCR (qRT-PCR) analysis.

| **Gene abbreviation** | **Full name** | **Primer sequence (5'→3')** | **Annealing temperature (℃)** |
| --- | --- | --- | --- |
| ***VlActin*** | Actin | F: GAGTGAGCCACACTGTTCCA  R: CGCGAACAATTTCCCGTTCA | 60 |
| ***VlADH*** | Alcohol dehydrogenase | F: GATCGCAGCGTAGAATGCAC  R: GGATGTCGCTTCTGGGCTTA | 60 |
| ***VlDHN1*** | Dehydrin 1 | F: CGGCTCACTTGGTCAGCAAT  R: TCCCACCCATT CCATCATCC | 60 |
| ***VlEDS1*** | Enhanced Disease Susceptibility 1 | F: TGCACGACATTGTACCTCGT  R: TAGATTACACGCCGCATGGC | 60 |
| ***VlERF*** | Ethylene Response Factor | F: GCTCCTATGACACGGTGGAA  R: TTCCTTGCATGTTTCGACGC | 60 |
| ***VlPOD*** | Peroxidase | F: CGTCCAATCAGGTTGACCCA  R: GATCCGACGTGAACAACCCT | 60 |
| ***VlWDS*** | Water Deficit Stress-related gene | F: AAGCGTAAAGAACACGTGGGA  R: GACGCTCGTGGAATGCATAG | 60 |
| ***VlpsbA*** | Photosystem II protein D1 gene | F: AGAGAGACGCGAAAGCGAAA  R: TGGAGGAGCAGCAATGAAGG | 60 |
| ***VlDREB1*** | Dehydration Response Element-Binding protein 1 | F: GGACTTAGCATCTCAGCCAACT  R: GAACCCTTAGCCGGAGCTTT | 60 |
| ***VlEPFL6*** | Epidermal Patterning Factor-Like 6 | F: TAGGTGGACCCGGATCATCA  R: GGCATGTACAACCTGTTGCC | 60 |
| ***VlrbcS*** | Ribulose-1,5-bisphosphate carboxylase/oxygenase small subunit | F: CTCAGGCCAATATGGTCGCA  R: GCTCGATGGTTAGGTCAGGG | 60 |

**Supplementary table S3.** Sample sizes for ln response ratio estimates in forest plots.

| **Figure panel** | **Trait** | **Sample size (n)** | **Data source** |
| --- | --- | --- | --- |
| Figure 5A | Total fresh weight | 20 | Final harvest 2024 |
|  | Shoot fresh weight | 20 | Final harvest 2024 |
|  | Root fresh weight | 20 | Final harvest 2024 |
|  | Total dry weight | 20 | Final harvest 2024 |
|  | Shoot dry weight | 20 | Final harvest 2024 |
|  | Root dry weight | 20 | Final harvest 2024 |
|  | Total water content | 20 | Final harvest 2024 |
|  | Shoot water content | 20 | Final harvest 2024 |
|  | Root water content | 20 | Final harvest 2024 |
|  | Root:shoot ratio | 20 | Final harvest 2024 |
|  | Inflorescence number | 20 | Final harvest 2024 |
|  | Inflorescence length | 20 | Final harvest 2024 |
|  | 100-seed weight | 5 | Seed sample 2024 |
|  | Seed length | 10 | Seed sample 2024 |
|  | Seed width | 10 | Seed sample 2024 |
| Figure 5B | Time taken for shoot appearance | 34 | March 2025 |
|  | Photosynthetic rate  (new shoot) | 9 | March 2025 |
|  | Stomatal conductance  (new shoot) | 9 | March 2025 |
|  | Plant height | 10 | August 2025 |
|  | Total fresh weight | 10 | August 2025 |
|  | Shoot fresh weight | 10 | August 2025 |
|  | Root fresh weight | 10 | August 2025 |
|  | Total dry weight | 10 | August 2025 |
|  | Shoot dry weight | 10 | August 2025 |
|  | Root dry weight | 10 | August 2025 |
|  | Total water content | 10 | August 2025 |
|  | Shoot water content | 10 | August 2025 |
|  | Root water content | 10 | August 2025 |
|  | Root:shoot ratio | 10 | August 2025 |
|  | Photosynthetic rate | 10 | August 2025 |
|  | Stomatal conductance | 10 | August 2025 |
|  | Fv/Fm | 10 | August 2025 |
| Figure 5C | *VlADH* | 4 | August 2025 |
|  | *VlDHN1* | 4 | August 2025 |
|  | *VlWDS* | 4 | August 2025 |
|  | *VlDREB1* | 4 | August 2025 |
|  | *VlEDS1* | 4 | August 2025 |
|  | *VlERF* | 4 | August 2025 |
|  | *VlPOD* | 4 | August 2025 |
|  | *VlpsbA* | 4 | August 2025 |
|  | *VlrbcS* | 4 | August 2025 |
|  | *VlEPFL6* | 4 | August 2025 |

**Supplementary table S4.** Monitored seasonal environmental conditions during irrigation treatment.

| **Environmental factor** | **Pre-rainy** | | **Rainy** | | **Post-rainy** | |
| --- | --- | --- | --- | --- | --- | --- |
|  | **Irrigated** | **Non-irrigated** | **Irrigated** | **Non-**  **irrigated** | **Irrigated** | **Non-**  **irrigated** |
| Soil water content (%) | 14.4±1.02^z^ | 10.1±0.97 | 27.6±1.62 | 24.8±1.50 | 13.4±2.10 | 11.3±0.25 |
| significance | *** | | *** | | *** | |
| Temperature (℃) | 20.9±0.53 | | 25.8±0.27 | | 28.2±0.23 | |
| Relative humidity (%) | 68.9±1.56 | | 85.1±1.36 | | 83.0±0.79 | |
| Light intensity  (μmol∙m^-2^∙s^-1^) | 869.4±61.20 | | 535.3±49.83 | | 555.2±31.08 | |

^z^ Values indicate standard error.
*** indicate significance at *P* < 0.001 by AVOVA (n=69 for pre-rainy; n=32 for rainy; n=39 for post- rainy).
